# Activation of Ventral Tegmental Area 5-HT_2C_ Receptors Reduces Incentive Motivation

**DOI:** 10.1038/npp.2016.264

**Published:** 2016-12-21

**Authors:** Lourdes Valencia-Torres, Cristian M Olarte-Sánchez, David J Lyons, Teodora Georgescu, Megan Greenwald-Yarnell, Martin G Myers, Christopher M Bradshaw, Lora K Heisler

**Affiliations:** 1 Rowett Institute of Nutrition and Health, University of Aberdeen, Aberdeen, UK; 2 Division of Metabolism, Endocrinology, and Diabetes, Department of Internal Medicine, University of Michigan, Ann Arbor, MI, USA; 3 Division of Psychiatry, University of Nottingham, Queen's Medical Centre, Nottingham, UK

## Abstract

Obesity is primarily due to food intake in excess of the body's energetic requirements, intake that is not only associated with hunger but also the incentive value of food. The 5-hydroxytryptamine 2C receptor (5-HT_2C_R) is a target for the treatment of human obesity. Mechanistically, 5-HT_2C_Rs are positioned to influence both homeostatic feeding circuits within the hypothalamus and reward circuits within the ventral tegmental area (VTA). Here we investigated the role of 5-HT_2C_Rs in incentive motivation using a mathematical model of progressive ratio (PR) responding in mice. We found that the 5-HT_2C_R agonist lorcaserin significantly reduced both *ad libitum* chow intake and PR responding for chocolate pellets and increased c-fos expression in VTA 5-HT_2C_R expressing γ-aminobutyric acid (GABA) neurons, but not 5-HT_2C_R expressing dopamine (DA) neurons. We next adopted a chemogenetic approach using a *5-HT*_*2C*_*R*^*CRE*^ line to clarify the function of subset of 5-HT_2C_ receptor expressing VTA neurons in the modulation of appetite and food-motivated behavior. Activation of VTA 5-HT_2C_ receptor expressing neurons significantly reduced *ad libitum* chow intake, operant responding for chocolate pellets, and the incentive value of food. In contrast, chemogenetic inhibition of VTA 5-HT_2C_ receptor expressing neurons had no effect on the feeding behavior. These results indicate that activation of the subpopulation of 5-HT_2C_R neurons within the VTA is sufficient to significantly reduce homeostatic feeding and effort-based intake of palatable food, and that this subset has an inhibitory role in motivational processes. These findings are relevant to the treatment of obesity.

## Introduction

The activity of the monoamine neurotransmitter 5-hydroxytryptamine (5-HT; serotonin) within the brain has a functional role in the regulation of feeding behavior and body weight (Blundell, 1986; Hill and Blundell, 1990), and as such has been a target for obesity medications (Halford *et al*, 2011; Vickers and Clifton, 2012). These effects of 5-HT are primarily achieved via action at the G-protein-coupled 5-HT_2C_ receptor (5-HT_2C_R) subtype, which is mainly, if not exclusively, expressed within the central nervous system. Activation of 5-HT_2C_Rs promotes weight loss by decreasing food intake (Martin *et al*, 2011; Higgs *et al*, 2016) through the modulation of the activity of neuronal pathways regulating energy balance, including those within the arcuate nucleus of the hypothalamus (Burke and Heisler, 2015). However, 5-HT_2C_Rs are also positioned to influence motivational circuits processing rewarding stimuli (Kranz *et al*, 2010). Specifically, 5-HT_2C_Rs within the ventral tegmental area (VTA) (Bubar and Cunningham, 2007; Bubar *et al*, 2011) exert an inhibitory effect upon the mesoaccumbens dopamine (DA) system (Di Matteo *et al*, 2000; Hayes *et al*, 2009) which has been implicated in motivational processes and effort-related choice (Salamone and Correa, 2013). Collectively, these findings suggest that 5-HT_2C_Rs may modulate feeding not solely by influencing energy homeostasis via the hypothalamus and functionally related structures, but also by regulating appetitive motivation (operant responding).

Several behavioral tasks have been used to assess motivation. One such task is the progressive ratio (PR) schedule, in which the number of responses required per reinforcer is progressively increased (Hodos, 1961). The ratio at which the subject stops responding (the ‘breakpoint') is regarded as a measure of motivation (Richardson and Roberts, 1996). The selective 5-HT_2C_R agonists lorcaserin ((1R)-8-chloro-1-methyl-2,3,4,5-tetrahydro-1H-3-benzazepine) (Meltzer and Roth, 2013), Ro-600175 ((αS)-6-chloro-5-fluoro-α-methyl-1H-indole-1-ethanamine) and CP-809101 (2-(3-chlorobenzyloxy)-6-(piperazin-1-yl)pyrazine); and non-selective 5-HTR agonists with high affinity for 5-HT_2C_R, such as mCPP (*m*-chlorophenylpiperazine) and MK-212 (6-chloro-2-(l-piperazinyl)pyrazine) reduce the breakpoint for food (Bezzina *et al*, 2015; Fletcher *et al*, 2009; Higgins *et al*, 2012), cocaine (Burbassi and Cervo, 2008, Harvey-Lewis *et al*, 2015), ethanol (Rezvani *et al*, 2014), and nicotine (Fletcher *et al*, 2012), effects that are blocked by 5-HT_2C_R antagonists (Bezzina *et al*, 2015; Fletcher *et al*, 2012). Thus, lorcaserin, recently approved by the US FDA for the treatment of human obesity, may also have therapeutic potential for nicotine (Higgins *et al*, 2012; Levin *et al*, 2011) and ethanol dependence (Rezvani *et al*, 2014). Although 5-HT_2C_R activation appears to have an inhibitory role in motivational processes (Filip *et al*, 2012; Fletcher *et al*, 2012), the subset of 5-HT_2C_ receptor expressing neurons underlying these effects has yet to be identified.

Here we investigated the role of 5-HT_2C_Rs in food motivation in mice. To this end, we used a mathematical model (Bradshaw and Killeen, 2012) derived from Killeen's general theory of schedule-controlled behavior, the Mathematical Principles of Reinforcement (MPR, Killeen, 1994), that enables the experimenter to discriminate between changes in motor capability and the incentive value of food in PR performance. First, we analyzed the effect of systemic administration of 5-HT_2C_R agonist lorcaserin, on home cage chow intake and operant responding for chocolate pellets. Next, we characterized the neurons expressing 5-HT_2C_Rs within the VTA and their response to lorcaserin as measured by c-fos immunoreactivity (IR). To investigate the role of VTA 5-HT_2C_ receptor expressing neurons in the modulation of appetite and the food-motivated behavior, we used a chemogenetic approach (designer receptors exclusively activated by a designer drug, DREADD) (Urban and Roth, 2015) to acutely activate or inhibit this population of neurons using a recently developed *5-HT*_*2C*_*R*^*CRE*^ mouse line (Burke *et al*, 2016). These studies identify the therapeutic potential of the activation of a small subset of 5-HT_2C_ receptor expressing neurons in the reduction of homeostatic feeding and also more broadly in incentive motivation.

## Materials and methods

### Subjects

*5-HT*_*2C*_*R*^*CRE*^ mice were intercrossed with ROSA26-stop-enhanced yellow fluorescent protein (YFP) (B6.129X1-*Gt(ROSA)26Sortm1(EYFP)Cos*/J; Jackson Laboratory) to create a *5-HT*_*2C*_*R*^*CRE:YPF*^ line (Burke *et al*, 2016). All the mice were group-housed and maintained on a 12 h light/dark cycle with *ad libitum* access to water and standard laboratory chow diet, unless otherwise stated. All the experiments were in accordance with guidelines and approvals of the U.K. Animals (Scientific Procedures) Act 1986 or the University of Michigan Committee on the Use and Care of Animals.

### Drugs

Lorcaserin HCl (3, 7, 10 or 12 mg/kg; LGM Pharma, Nashville, TN) and clozapine-N-oxide (CNO) (2 mg/kg; Sigma-Aldrich, Gillingham, UK) were dissolved in 0.9% NaCl and injected intraperitoneally (i.p.) at a volume of 10 ml/kg body weight.

### Stereotaxic Viral Vector Injection

Male and female *5-HT*_*2C*_*R*^*CRE:YFP*^mice were injected bilaterally with 0.25 μl AAV-hSyn-DIO-hM3D(G_q_)-mCherry, AAV-hSyn-DIO-hM4D(G_i_)-mCherry or AAV-DIO-mCherry (University of North Carolina Vector Core Facilities, Chapel Hill, NC) into the VTA (stereotaxic coordinates, millimeters from bregma: antero-posterior −3.16  medio-lateral ±0.56; dorso-ventral −4.5) using a stereotaxic frame (see Supplementary Information for further details). We did not observe significant sex differences between treatment responses to food intake (*5-HT*_*2C*_*R*^*CRE:YPF*^::hM3D_q_-mCherry^VTA^: *n*=14; F_1,10_=2.78; NS; *5-HT*_*2C*_*R*^*CRE:YPF*^::hM4D_i_-mCherry^VTA^: *n*=14; F_1,10_=1.12; NS) or the breakpoint in the *ad libitum* fed (*5-HT*_*2C*_*R*^*CRE:YPF*^::hM3D_q_-mCherry^VTA^: *n*=15; F_1,11_=1.36; NS; *5-HT*_*2C*_*R*^*CRE:YPF*^::hM4D_i_-mCherry^VTA^: *n*=29; F_1,25_=0.440; NS) or food-deprived conditions (*5-HT*_*2C*_*R*^*CRE:YPF*^::hM3D_q_-mCherry^VTA^: *n*=15; F_1,11_=0.216; NS; *5-HT*_*2C*_*R*^*CRE:YPF*^::hM4D_i_-mCherry^VTA^: *n*=30; F_1,26_=0.533; NS) and thereby combined data with male and female mice.

### Operant Conditioning

The mice were individually housed and trained on a PR schedule based on an exponential progression derived from the formula (5  ×  e^0.2*n*^)−5, rounded to the nearest integer, where *n* is the position in the ratio sequence (Richardson and Roberts, 1996; see Supplementary Information for further details). The breakpoint was defined as the last ratio completed before 5 min elapsed without any responding or, when this criterion was not met within the session, the highest completed ratio (Olarte-Sanchez *et al*, 2012). The mathematical model (Bradshaw and Killeen, 2012) used to analyze PR performance comprises three key equations. The parameters of these equations provide separate numerical indices of the motivational impact of the reinforcer and the motor capability of the organism. The linear waiting equation (Wynne *et al*, 1996) is used to predict the post-reinforcement pause in each ratio (*T*_P,*i*_) from the time taken to complete the preceding ratio (total time, *T*_TOT,*i*−1_):


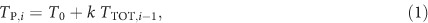


where *T*_0_ is the initial post-reinforcement pause and *k* is the slope of the linear waiting function. Two further equations define running response rate, *R*_RUN_, and overall response rate, *R*_OVERALL_, in successive ratios of the schedule:


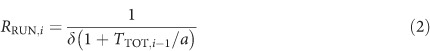



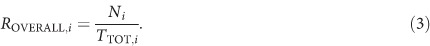


The parameters *δ* and *a* are the fundamental ‘motor' and ‘motivational' parameters of the model: *δ* expresses the minimum time needed to execute a response, and *a* the duration of behavioral activation induced by a single reinforcer (Bradshaw and Killeen, 2012; Killeen, 1994; see Supplementary Information for further details).

### Food Intake

The mice were individually housed and habituated for 5 days in TSE Phenomaster chambers (TSE, Bad Homburg, Germany) that record food intake automatically using weight sensors attached to food containers suspended from the ceiling. On each treatment day, 30 min before the onset of the dark cycle, the food hopper was automatically closed and the mice were injected with treatment (saline, lorcaserin or CNO). At the onset of the dark cycle, the food hopper was opened and chow intake was recorded for the next hour.

### Immunohistochemistry

Immunohistochemistry (IHC) was performed on coronally sectioned 4% paraformaldehyde fixed brain tissue using methods previously reported (Burke *et al*, 2016) using the following primary antibodies to detect immunoreactivity (IR) for *c-fos* (#2672548, 1:5000, Millipore) or immunofluorescence (IF) for 5-HT_2C_R (#L1813, 1:300; Santa Cruz Biotechnology), *c-fos* (#L1610; 1:800, Santa Cruz Biotechnology), γ-aminobutyric acid (GABA) synthesizing enzyme glutamic acid decarboxylase (GAD; #J2804; 1:150, Santa Cruz Biotechnology), the dopamine (DA) synthesizing enzyme tyrosine hydroxylase (TH; #2716631; 1:1000, Millipore), green fluorescent protein (GFP; #GR279236-1; 1: 800, Abcam), or mCherry (#31089; 1:800, Rockland). Images were acquired using an Axioskop II microscope (Carl Zeiss, Germany), processed with Adobe Photoshop (Adobe Systems Software, Ireland) and analyzed with Image J software (NIH). For IHC quantification analysis, the VTA was defined using the Mouse Brain Atlas (Paxinos and Franklin, 2001) and sections containing the VTA (from bregma −2.92 to −3.88 mm) were counted bilaterally (see Supplementary Information for further details).

### Electrophysiology

Egith-to-twelve–week-old *5-HT*_*2C*_*R*^*CRE:YFP*^ mice were deeply and terminally anesthetized with sodium pentobarbital and then decapitated. The brain was rapidly removed and placed in an ice-cold oxygenated (95%O_2_/5%CO_2_) high sucrose ‘slicing' solution. Coronal slices containing the VTA were prepared and immediately transferred to a ‘recording' solution in a continuously oxygenated holding chamber at 35°C for a period of 25 min. Subsequently, the slices were allowed to recover in the ‘recording' solution at room temperature for a minimum of 1 h before recording. All patch clamp recordings were made using multiclamp 700B amplifier, and the data were filtered at 2 kHz and digitized at 10 kHz (see Supplementary Information for further details).

### Statistics

For lorcaserin treatment PR and food intake experiments, the weight of the food consumed, the estimates derived from the mathematical model and the breakpoint were analyzed using analysis of variance (ANOVA) with repeated measures for treatment (lorcaserin dose) with Dunnett's *post hoc* test. For the chemogenetic food intake and PR experiments, the weight of the food consumed, estimates derived from the mathematical model and the breakpoint were analyzed using ANOVA (group [AAV-hM3D(Gq)), AAV-mCherry] × treatment [vehicle, CNO]) or (group [AAV-hM4D(Gi)), AAV-mCherry] × treatment [vehicle, CNO]) with repeated measures for treatment, and *post hoc* comparisons using Tukey's test. A significance criterion of *p*<0.05, two-tailed, was adopted in all the statistical analyses.

## Results

### 5-HT_2C_R Agonist Lorcaserin Reduces Food Intake and Decreases Operant Responding for Food Reward

**Figure 1:**
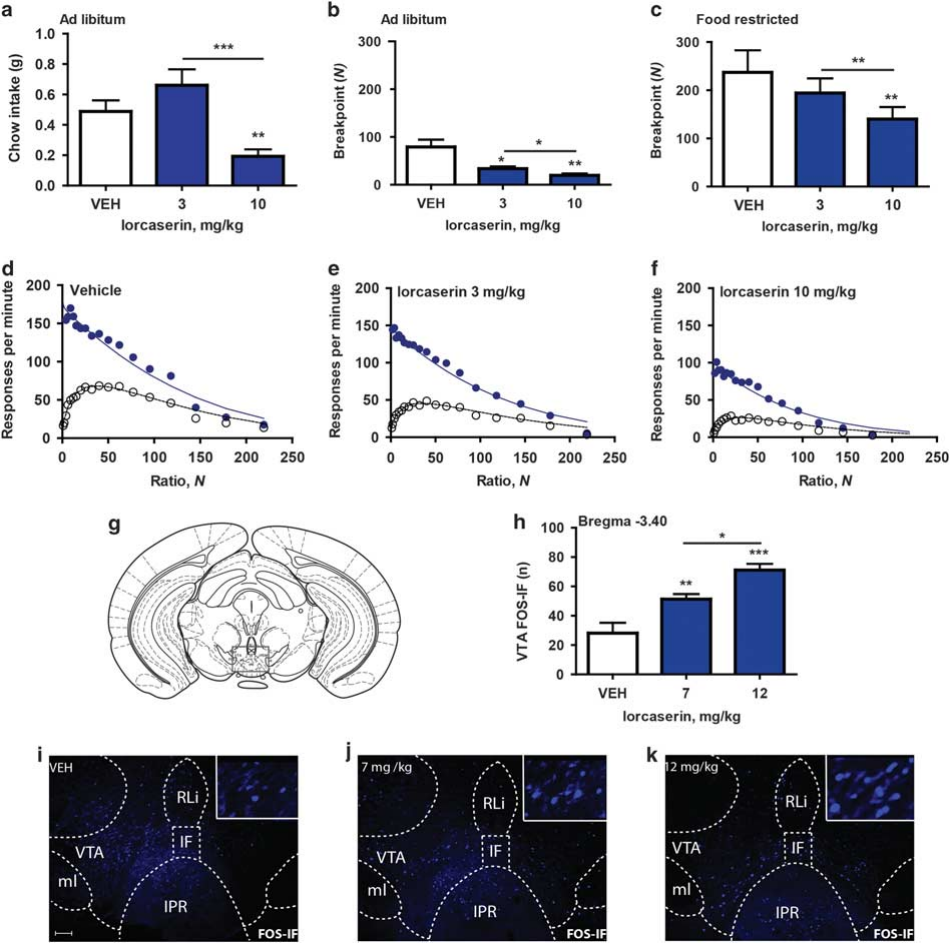
Effect of lorcaserin on food intake and operant responding. (a) Lorcaserin 10 mg/kg reduced 1 h chow intake (*n*=14). (b) Lorcaserin 3 and 10 mg/kg reduced the breakpoint when mice were in *ad libitum* condition (*n*=9). (c) Lorcaserin 10 mg/kg reduced the breakpoint when mice were in food-restricted condition (*n*=9). (d–f) Effect of lorcaserin on performance on the progressive-ratio schedule. Ordinate, response rate; abscissa, response/reinforcer ratio, *N*. Points are group mean data: unfilled symbols indicate running response rate, filled symbols indicate overall response rate. The curves are best-fit functions defined by equations (2) and (3). (g) Schematic of the location of the VTA defined by the Mouse Brain Atlas (Paxinos and Franklin, 2001). (h) Lorcaserin 7 and 12 mg/kg increased FOS-IF (blue) within the VTA (*n*=6–7 per group). (i–k) FOS-IF (blue) within the VTA following administration of vehicle, lorcaserin 7 or 12 mg/kg (scale bar: 100 μm). All the data are presented as mean±SEM. ^∗^*p*<0.05; ^∗∗^*p*<0.01; ^∗∗∗^*p*<0.001.

Consistent with previous reports (Fletcher *et al*, 2009; Thomsen *et al*, 2008; Burke *et al*, 2014), lorcaserin significantly reduced home cage *ad libitum* chow intake compared with saline (*n*=14; F_2,26_=11.76; *p*<0.001; Figure 1a). To assess the effect of lorcaserin on food-motivated behavior, the mice were trained on a PR schedule and tested during two conditions: *ad libitum* fed and food restricted. In the *ad libitum* fed condition, lorcaserin 3 and 10 mg/kg significantly reduced the breakpoint compared with vehicle (*n*=9; F_2,16_=11.76; *p*<0.001; Figure 1b). The breakpoint was also reduced by 10 mg/kg lorcaserin during the food restricted condition (*n*=9; F_2,16_=10.99; *p*<0.01; Figure 1c). Further analysis of performance on the PR schedule during the food-restricted condition indicated that *R*_RUN_ declined monotonically towards zero, whereas *R*_OVERALL_ rose to a peak before declining towards zero. In accordance with the mathematical model, response rates conformed closely to equations (2) and (3) (*R*^2^=0.981 [vehicle], 0.990 [lorcaserin 3 mg/kg], and 0.978 [lorcaserin 10 mg/kg]) and post-reinforcement pause duration in successive ratios was linearly related to the prior inter-reinforcer interval, as indicated by values of *R*^*2*^ above 0.9 (Figures 1d–f). Lorcaserin 3 and 10 mg/kg significantly increased the value of *k* (*n*=9; F_2,16_=14.46; *p*<0.0001; Supplementary Figure S1D), while having no significant effect on the ‘motivational' parameter, *a*, (*n*=9; F_2,16_=0.31; NS; Supplementary Figure S1A) or *T*_0_ (*n*=9; F_2,16_=0.93; NS; Supplementary Figure S1C). The ‘motor' parameter, *δ*, was increased by lorcaserin, the effect of 10 mg/kg being statistically significant (*n*=9; F_2,16_=19.6; *p*<0.0001; Supplementary Figure S1B). These data suggest that lorcaserin significantly reduces operant responding for food reward by impacting motor processes.

### Lorcaserin Increases the Activity of VTA Neurons

The VTA is a structure involved in motivation, food intake and motor function. To determine whether lorcaserin influences the activity of VTA neurons, the mice were treated with vehicle or lorcaserin (7 or 12 mg/kg) and brains were processed for FOS-IF. First, it was confirmed that 7 or 12 mg/kg of lorcaserin reduced chow intake (*n*=7–8 per group; F_2,19_=8.12; *p*<0.001; Supplementary Figure S2A) and the breakpoint in both the *ad libitum* (*n*=25; F_2,48_=41.65; *p*<0.0001; Supplementary Figure S2B) and food-restricted conditions (*n*=25; F_2,48_=23.58; *p*<0.0001; Supplementary Figure S2C). Next, we assessed VTA FOS-IF and observed that lorcaserin significantly increased FOS-IF compared with vehicle (*n*=6–7 per group; F_2,17_=17.27; *p*<0.0001; Figures 1g–k). These data indicate that doses of lorcaserin that reduced food intake and operant responding increase the activity of VTA neurons.

### Lorcaserin Increases the Activity of VTA 5-HT_2C_R Expressing GABA-Ergic Neurons

**Figure 2:**
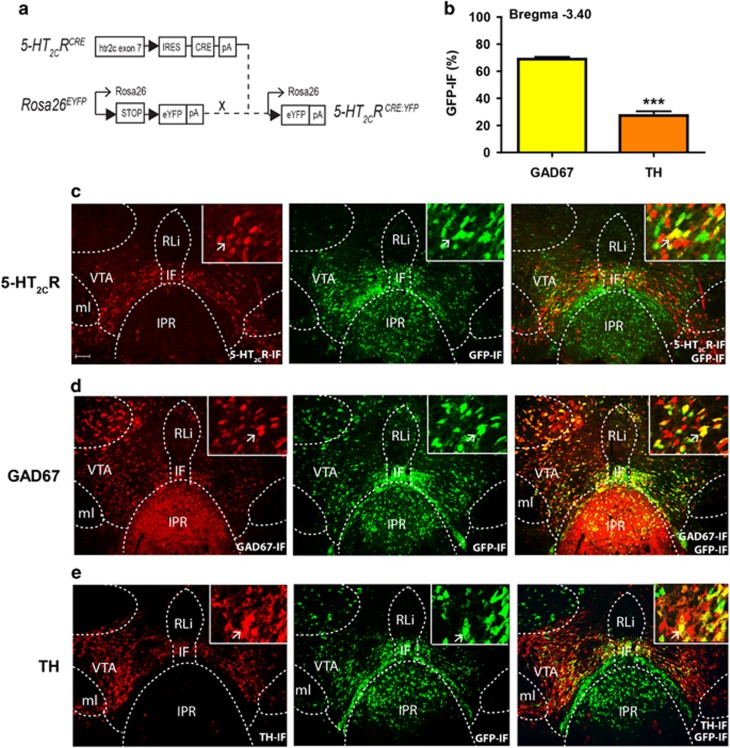
*5-HT*_*2C*_*R*^*CRE:YFP*^ VTA neurons co-express GABA and dopamine. (a) A *5-HT*_*2C*_*R*^*CRE*^ mouse line in which Cre recombinase is driven by a 5-HT_2C_R promoter was intercrossed with a ROSA26-stop-EYFP reporter mouse to generate a *5-HT*_*2C*_*R*^*CRE:YFP*^ line to facilitate the visualization of VTA 5-HT_2C_R expressing cells. (b) VTA *5-HT*_*2C*_*R*^*CRE:YFP*^ containing neurons (GFP-IF) have a greater co-expression with GAD67-IF compared with TH-IF (*n*=4 per group, *p*<0.001). (c) VTA *5-HT*_*2C*_*R*^*CRE:YFP*^ (GFP-IF; green) are co-localized (overlay, yellow) with endogenous 5-HT_2C_R-IF (red). (d) VTA *5-HT*_*2C*_*R*^*CRE:YFP*^ (GFP-IF; green) containing neurons and GAD67 (red) are co-localized (yellow). (e) VTA *5-HT*_*2C*_*R*^*CRE:YFP*^ (GFP-IF; green) containing neurons and TH (red) are co-localized (yellow). All the data are presented as mean±SEM. ^∗∗∗^*p*<0.001. Scale bar: 100 μm.

To characterize the neurochemical identity of 5-HT_2C_R expressing VTA neurons, we intercrossed a *5-HT*_*2C*_*R*^*CRE*^ line with a *ROSA26*^*EYFP*^ line (Figure 2a) to generate *5-HT*_*2C*_*R*^*CRE:YPF*^mice. In agreement with previous reports of endogenous 5-HT_2C_R expression (Bubar and Cunningham, 2007; Bubar *et al*, 2011), we observed that *5-HT*_*2C*_*R*^*CRE:YFP*^positive cells were widely expressed within the VTA and co-localized with 5-HT_2C_R-IF (Figure 2c). To explore the neurochemical phenotype of VTA 5-HT_2C_R expressing neurons, we performed dual-IHC for GPF (5-HT_2C_R) and GAD67 (GABA) or TH (dopamine). In line with previous reports using a 5-HT_2C_R antibody (Bubar and Cunningham, 2007; Bubar *et al*, 2011), we found that *5-HT*_*2C*_*R*^*CRE:YPF*^is expressed with both GAD67 and TH neurons (Figures 2d and e). Specifically, we observed that the majority of VTA *5-HT*_*2C*_*R*^*CRE:YPF*^expressing neurons were GAD67 positive (69%), whereas a significantly smaller percentage co-expressed TH (27%) (*n*=3 per group; *t*(4)=11.69; *p*<0.001; Figure 2b and Supplementary Figure S3). These data suggest that 5-HT_2C_Rs are predominantly positioned to influence the activity of GABAergic VTA neurons.

**Figure 3:**
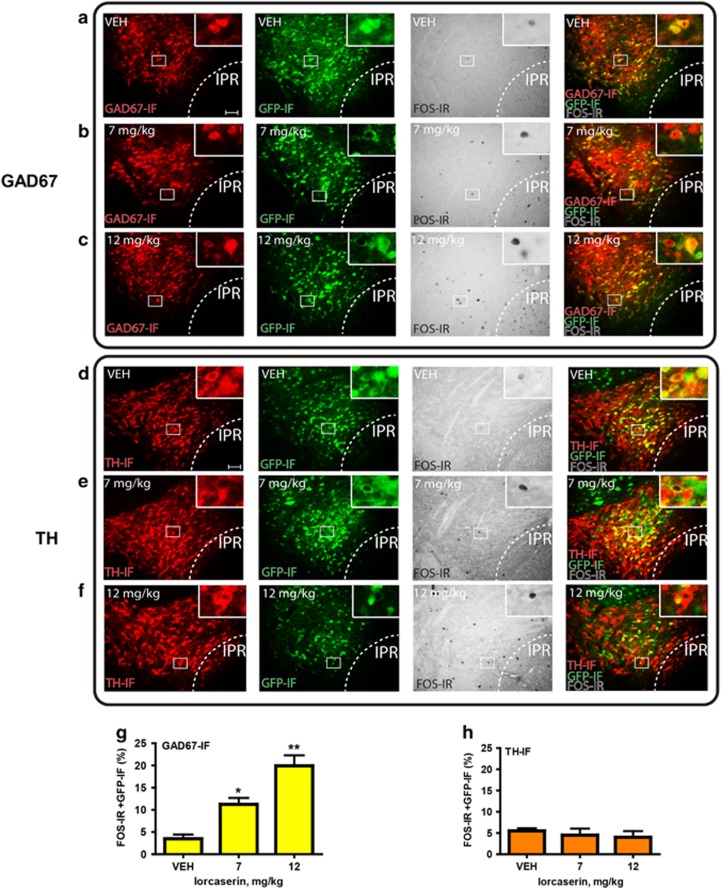
Lorcaserin increases c-Fos in VTA 5-HT_2C_R GABAergic, but not dopamine neurons. (a-c) c-Fos expression (FOS-IR, gray) in VTA 5-HT_2C_R containing neurons (GFP-IF, green) co-expressing GAD67 (GAD67-IF, red) in *5-HT*_*2C*_*R*^*CRE:YFP*^ mice following (a) vehicle, (b) lorcaserin 7 mg/kg or (c) 12 mg/kg administration. (d–f) c-Fos expression (FOS-IR, gray) in VTA 5-HT_2C_R containing neurons (GFP-IF, green) co-expressing TH (TH-IF, red) in *5-HT*_*2C*_*R*^*CRE:YFP*^ mice following (d) vehicle, (e) lorcaserin 7 mg/kg or (f) 12 mg/kg administration. (g) Lorcaserin dose-dependently increases FOS-IR in GAD67-positive 5-HT_2C_R expressing neurons (*n*=4 per group, *p*<0.001), but not in (h) TH-positive 5-HT_2C_R expressing neurons (*n*=4 per group, NS). All the data are presented as mean±SEM. ^∗^*p*<0.05; ^∗∗^*p*<0.01. Scale bar: 50 μm.

We next evaluated the neurochemical phenotype of 5-HT_2C_R expressing neurons responsive to lorcaserin treatment. *5-HT*_*2C*_*R*^*CRE:YFP*^ mice were treated with vehicle or lorcaserin (7 or 12 mg/kg) and tissue processed for triple-IHC for GFP, FOS and GAD67 or TH. Lorcaserin significantly and dose-dependently increased the activity (FOS-IR) of GAD67-positive 5-HT_2C_R expressing VTA neurons (*n*=3 per group; F_2,6_=23.3; *p*<0.001; Figures 3a–c and g), but had no effect on TH-positive 5-HT_2C_R expressing VTA neurons (*n*=4 per group; F(2,9)=0.52; NS; Figures 3d–f and h). These findings suggest that lorcaserin at doses that reduce food intake and PR responding for food reward increase the activity of VTA GABAergic, but not dopaminergic neurons.

### Chemogenetic Activation of VTA 5-HT_2C_R Expressing Neurons Reduce Food Intake and Operant Responding

**Figure 4:**
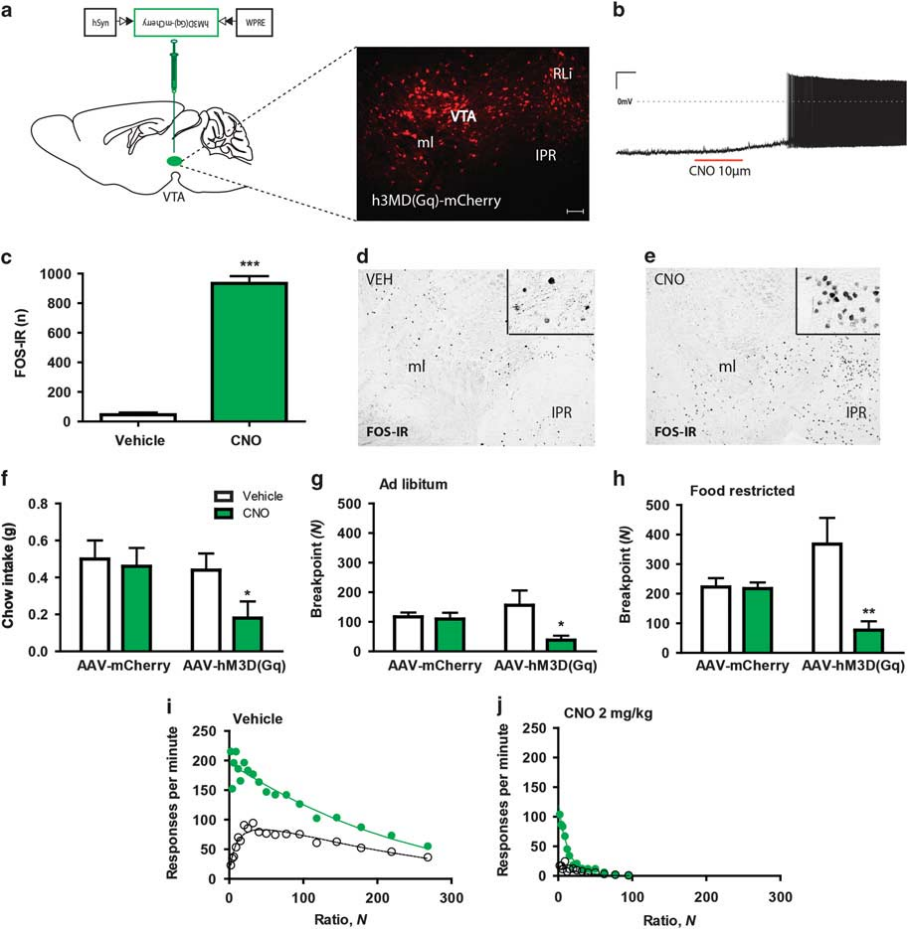
Chemogenetic activation of VTA 5-HT_2C_R neurons reduces food intake and operant responding for food reward. (a) Representative image of Cre-dependent expression of hM3Dq-mCherry specifically within the VTA of a *5-HT*_*2C*_*R*^*CRE:YFP*^ mouse. (b) Firing rate of *5-HT*_*2C*_*R*^*CRE:YPF*^::hM3D_q_-mCherry^VTA^ neurons increased upon 10 μM CNO application. (c–e) CNO 2 mg/kg increased FOS-IR within *5-HT*_*2C*_*R*^*CRE:YPF*^::hM3D_q_-mCherry^VTA^ compared with vehicle (*n*=3 per group). (f) CNO 2 mg/kg reduced 1 h chow intake (*n*=14, *p*<0.05). (g) CNO 2 mg/kg reduced the breakpoint in the experimental group [AAV-hM3D (G_q_)] compared with control [AAV-mCherry] in *ad libitum* condition (*n*=15) and (h) food-restricted condition (*n*=15). (i and j) Effect of chemogenetic activation of VTA 5-HT_2C_R neurons on performance on a PR schedule. Ordinate, response rate; abscissa, response/reinforcer ratio, *N*. Points are group mean data: unfilled symbols indicate running response rate, filled symbols indicate overall response rate. The curves are best-fit functions defined by equations (2) and (3). All the data are presented as mean±SEM. ^∗^*p*<0.05; ^∗∗^*p*<0.01; ^∗∗∗^*p*<0.001. Scale bar: 100 μm.

We next considered whether selective activation or inhibition of VTA 5-HT_2C_R expressing neurons was able to influence *ad libitum* food intake or operant responding for food reward. To investigate this, *5-HT*_*2C*_*R*^*CRE:YPF*^mice received bilateral VTA injections with AAVs that mediate the Cre-dependent expression of designer receptors exclusively activated by designer drugs (DREADDs; expressed as DREADD-mCherry fusion proteins, hM3Dq; *5-HT*_*2C*_*R*^*CRE:YPF*^::hM3D_q_-mCherry^VTA^) (Figure 4a). DREADDs are designer muscarinic receptor variants that can only be activated by an otherwise biologically inert designer drug, CNO (Alexander *et al*, 2009). We first confirmed that DREADD-fused mCherry reporter protein was expressed in the VTA (Figure 4a). Next, we demonstrated that CNO activated *5-HT*_*2C*_*R*^*CRE:YPF*^::hM3D_q_-mCherry^VTA^ cells with whole-cell electrophysiological recordings in *ex vivo* VTA slices (*n*=4/4, 100% responsive; Figure 4b) and *in vivo* using FOS-IR (*n*=3 per group; *t*(4)=17.45; *p*<0.0001; Figures 4c–e).

Subsequently, we examined the effect of chemogenetic activation of VTA 5-HT_2C_R expressing neurons on feeding behavior. The administration of CNO significantly reduced *ad libitum* chow intake compared with vehicle in the home cage in *5-HT*_*2C*_*R*^*CRE:YPF*^::hM3D_q_-mCherry^VTA^ mice (*n*=14; F_1,12_=5.47; *p*<0.05; Figure 4f). These data suggest that activation of VTA 5-HT_2C_R expressing neurons is sufficient to significantly reduce food intake. To assess the effect of activation of VTA 5-HT_2C_R expressing neurons on food-motivated behavior, *5-HT*_*2C*_*R*^*CRE:YPF*^::hM3D_q_-mCherry^VTA^ mice were trained on a PR schedule. The administration of CNO significantly reduced the breakpoint in both the *ad libitum* fed (*n*=15; F_1,13_=5.11; *p*<0.05; Figure 4g) and food-restricted (*n*=15; F_1,13_=21.5, *p*<0.001; Figure 4h) conditions in *5-HT*_*2C*_*R*^*CRE:YPF*^::hM3D_q_-mCherry^VTA^ mice compared with vehicle treatment. Further analysis revealed that *R*_RUN_ declined monotonically towards zero, whereas *R*_OVERALL_ rose to a peak before declining towards zero. In accordance with the mathematical model, response rates conformed closely to equations (2) and (3) (control group [AAV-mCherry]: *R*^2^=0.960 [vehicle], 0.947 [CNO 2 mg/kg] experimental group [AAV-hM3D (Gq)]: *R*^2^=0.961 [vehicle], 0.979 [CNO 2 mg/kg]) (Figures 4i–j). Quantitative analysis indicated a reduction of *a* (*n*=15; F_1,13_=6.6, *p*<0.05), but no change in *δ* (*n*=15; F_1,13_= 1.70; NS). These results are consistent with a reduction in the incentive value of the reinforcer without a concomitant impairment of motor competence (Supplementary Figure S2A and B). Activation of VTA 5-HT_2C_R expressing neurons significantly increased the value of *k* (*n*=15; F_1,13_=12.6; *p<*0.01; Supplementary Figure S2D), while having no effect on *T*_0_ (*n*=15; F_1,13_=1.3; NS; Supplementary Figure S2C). Collectively, these data indicate that the selective activation of VTA 5-HT_2C_R expressing neurons is sufficient to significantly reduce home cage food intake and operant responding for food reward, without altering motor performance.

**Figure 5:**
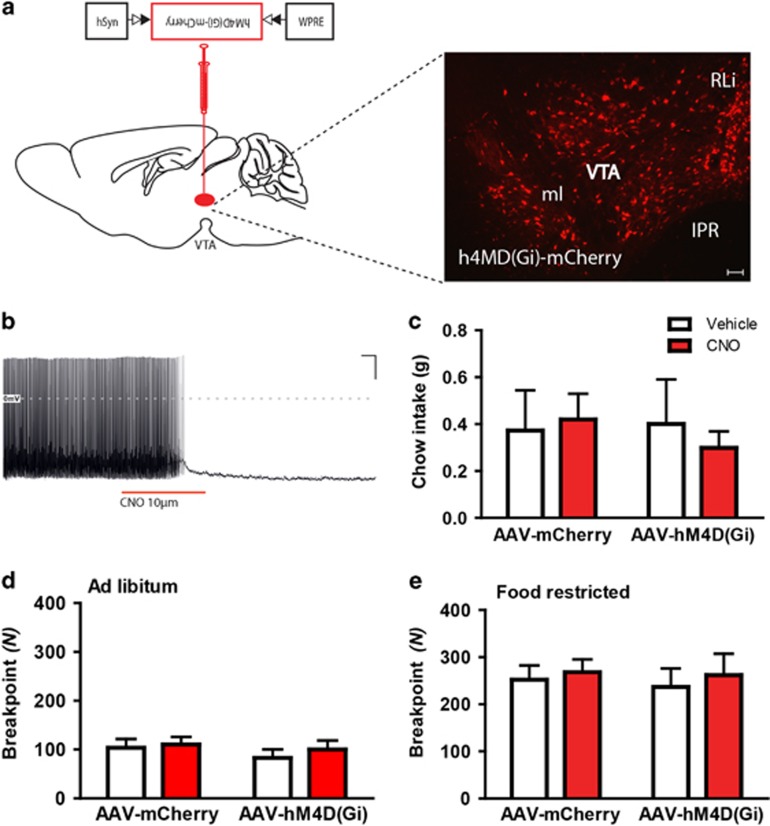
Chemogenetic inhibition of VTA 5-HT_2C_R neurons does not alter food intake or operant responding for food reward. (a) Representative image of Cre-dependent expression of hM4D(Gi)-mCherry specifically within the VTA of a 5-HT_2C_R^CRE:YFP^ mouse. (b) Firing rate of *5-HT*_*2C*_*R*^*CRE:YPF*^::hM4D_i_-mCherry^VTA^ neurons decreased upon 10 μM CNO application. (c) CNO 2 mg/kg had no effect on 1 h chow intake (*n*=14). (d) CNO 2 mg/kg had no effect on the breakpoint in the experimental group [AAV-hM4D (G_i_)] compared with the control [AAV-mCherry] in *ad libitum* condition (*n*=29) or (e) food-restricted condition (*n*=30).

To investigate whether VTA 5-HT_2C_R expressing neurons are required for normal food intake and reward processing, we chemogenetically silenced VTA 5-HT_2C_R expressing cells. Specifically, *5-HT*_*2C*_*R*^*CRE:YPF*^mice received bilateral VTA injections with AAVs that mediate the Cre-dependent expression of inhibitory (G_i_) DREADD-mCherry fusion protein, hM4D_i_; *5-HT*_*2C*_*R*^*CRE:YPF*^::hM4D_i_-mCherry^VTA^ (Figure 5a). First, we performed patch-clamp recordings in *ex vivo 5-HT*_*2C*_*R*^*CRE:YPF*^::hM4D_i_-mCherry^VTA^ slices to confirm the inhibitory effect of CNO on cell activity. Bath application of CNO reduced firing rate in 8/8 *5-HT*_*2C*_*R*^*CRE:YPF*^::hM4D_i_-mCherry^VTA^ expressing neurons (Figure 5b). The effect of CNO administration on homeostatic food intake and food-motivated behavior in *5-HT*_*2C*_*R*^*CRE:YPF*^::hM4D_i_-mCherry^VTA^ mice was then assessed. Treatment with CNO had no effect on home cage chow intake (*n*=14; F_1,12_= 0.33; NS; Figure 5c) or PR responding for food reward in *ad libitum* (*n*=29; F_1,27_= 1.93; NS; Figure 5d) or food-restricted conditions (*n*=30; F_1,28_=0.05; NS; Figure 5e) compared with vehicle.

Though this chemogenetic approach suggests that reducing the activity of VTA 5-HT_2C_R expressing neurons via *5-HT*_*2C*_*R*^*CRE:YPF*^::hM4D_i_-mCherry^VTA^ is not sufficient to alter normal food intake or motivation for food reward, targeted genetic knockdown of VTA 5-HT_2C_Rs is required to establish the specific function of VTA 5-HT_2C_Rs in food intake and motivation for food reward. However, of translational relevance to human obesity treatment, we observed that activation of this discrete subset of VTA neurons is sufficient to produce a significant effect on multiple types of feeding behavior. These include, *ad libitum* food intake in the home cage and the PR task where the incentive value of food reward is manipulated by food-deprivation levels. Activation of this small subset of 5-HT_2C_R expressing neurons produced a consistent reduction in the feeding behavior that was not associated with changes in motor performance, suggesting that the effect is directly related to incentive motivation.

## Discussion

Due to its prevalence and deleterious health consequences, obesity is one of the primary global healthcare challenge of the 21st century. Discerning a new means to combat the obesity epidemic is therefore of paramount clinical importance. Obesity is primarily due to the consumption of food in excess of the body's energetic requirements, energy that is then stored as fat. 5-HT, via action at 5-HT_2C_Rs, produces a potent inhibitory effect on the feeding behavior. Capitalizing on this effect, the 5-HT_2C_R agonist lorcaserin, an obesity medication recently approved by the US FDA, reduces body weight by decreasing *ad libitum* food intake (Martin *et al*, 2011) and promoting satiety (Higgs *et al*, 2016).

However, as 5-HT_2C_Rs are expressed throughout the brain, precisely where and how lorcaserin exerts its effects upon feeding behavior has not been fully defined. Here we investigated a specific subset of 5-HT_2C_R expressing neurons that are sufficient to reduce food intake and moreover, food-motivated behavior. In particular, we were interested in food motivation because the drive or ‘motivation' to consume food in the absence of hunger is a primary behavior underpinning the development of obesity. Targeting and reducing this specific aspect of feeding behavior would be particularly attractive for the treatment of obesity.

### Obesity Medication Lorcaserin Reduces Food Intake and Operant Responding for Food Reward

Previous research has focussed on the role of 5-HT_2C_Rs in the modulation of homeostatic feeding by influencing the activity of the hypothalamic melanocortin system (Burke *et al*, 2016; Doslikova *et al*, 2013; Heisler *et al*, 2002; Lam *et al*, 2008; Xu *et al*, 2008). Here we observed that lorcaserin produced the expected reduction in home cage *ad libitum* intake of standard laboratory chow. Given that 5-HT_2C_Rs are also expressed in the VTA (Bubar and Cunningham, 2007; Bubar *et al*, 2011), a brain region associated with food and drug reward (DiLeone *et al*, 2012), we investigated lorcaserin's effects on a PR schedule using a mathematical model (Bradshaw and Killeen, 2012) derived from Killeen's Mathematical Principles of Reinforcement (MPR, Killeen, 1994). In agreement with previous reports in rats (Bezzina *et al*, 2015; Bradshaw and Killeen, 2012; Olarte-Sanchez *et al*, 2013, 2015; Valencia-Torres *et al*, 2014), our results indicate that operant behavior maintained by PR schedules is well described by the mathematical model in mice (Bradshaw and Killeen, 2012). Progressive ratio schedules are widely used to assess the effects of neuropharmacological interventions on motivation. This practice is based on the assumption that the traditional index of performance, the breakpoint, provides a quantitative index of motivation, or the incentive value of the reinforcer (Hodos, 1961; see Killeen *et al*, 2009). However, there is considerable evidence that the breakpoint is sensitive to motor as well as motivational factors (Aberman *et al*, 1998; Skjoldager *et al*, 1993). The mathematical model incorporates separate parameters that represent motivational and motor processes (*a* and δ, respectively); a change in the value of either parameter may give rise to a change in the breakpoint (Bradshaw and Killeen, 2012). Our findings indicated that systemically administered lorcaserin increased the value of *δ* without affecting *a.* The present finding is consistent with the results of Bezzina *et al* (2015) that showed that a reduction of the breakpoint induced by 5-HTR agonists mCPP and Ro-600175 was associated not with a reduction of the value of *a*, but with an increase of the value of *δ*, which could be reversed by 5-HT_2C_R antagonist SB-242084. These findings suggest that systemic treatment with 5-HT_2C_R agonists suppress operant performance via a detrimental effect on motor processes, thereby confounding interpretations of effects on motivational processes.

### Lorcaserin Increases the Activity of VTA 5-HT_2C_R GABAergic Neurons

Given that 5-HT_2C_Rs are expressed in multiple brain regions where they are positioned to perform different functions (including motor functions), we were interested in discerning a specific subset of 5-HT_2C_Rs capable of modulating food motivation without interfering with motor function. We observed that doses of lorcaserin that reduce food intake and PR responding increase the activity of VTA neurons. As the VTA neuronal population is phenotypically heterogeneous, we characterized the neurochemical phenotype of 5-HT_2C_R expressing neurons and their response to lorcaserin using of a new mouse line (*5-HT*_*2C*_*R*^*CRE:YFP*^; Burke *et al*, 2016). We observed that VTA 5-HT_2C_R expressing neurons were mainly co-expressed with GABA and a much smaller percentage were co-expressed with dopamine. Consistent with this anatomical localization, lorcaserin significantly and dose-dependently increased the activity of GABA-ergic neurons. This is consistent with the notion that activation of 5-HT_2C_ receptor expressing neurons of the VTA predominantly stimulates GABA-ergic neurons thereby suppressing the dopaminergic output of this structure (Filip *et al*, 2012; Navailles *et al*, 2008).

### Selective Chemogenetic Activation of VTA 5-HT_2C_R Expressing Neurons Reduces Food Intake and Operant Responding for Food Reward

Next, using a chemogenetic approach (Urban and Roth, 2015), we investigated the specific function of 5-HT_2C_R expressing cells within the VTA. Here we observed that selective activation of VTA 5-HT_2C_ receptor expressing neurons was sufficient to significantly reduce *ad libitum* chow intake (59%) to a degree comparable with systemic lorcaserin treatment (60%).

Next, we explored the role of VTA 5-HT_2C_ receptor expressing neurons in food-motivated behavior. We observed that chemogenetic activation of VTA 5-HT_2C_ receptor expressing neurons caused suppression of responding in the PR schedule and a reduction in the breakpoint both when mice were food restricted and fed *ad libitum*. Further quantitative analysis revealed a reduction of *a* but no change in *δ*, consistent with a reduction of the incentive value of the reinforcer with no concomitant impairment of motor competence. This is in line with previous research that demonstrated that 5-HT_2C_Rs within the VTA exert an inhibitory influence upon the mesoaccumbens dopamine (DA) system (Navailles *et al*, 2008) attenuating the effort-based intake of food (Salamone and Correa, 2013). Systemic administration of lorcaserin may engage additional systems that mask the specific motivational effect seen with acute stimulation of VTA 5-HT_2C_ receptor expressing neurons. For example, 5-HT_2C_Rs are expressed in several structures within the basal ganglia, where they exert influence over extrapyramidal motor functions (Graves *et al*, 2013; Miguelez *et al*, 2014).

Although activation of VTA 5-HT_2C_R expressing neurons produced a potent reduction in the feeding behavior, inhibition of these neurons did not alter food intake or PR responding for food reward. Targeted genetic knockdown studies of VTA 5-HT_2C_Rs are required to establish the necessity of this subpopulation on the regulation of food intake and motivation for food reward.

## Summary

Here we identify a new subpopulation of 5-HT_2C_Rs that are capable of reducing homecage food intake and food-motivated behavior without interfering with motor function, findings that reveal a new site of 5-HT_2C_R activation to pharmacologically exploit in the development of novel obesity medication. Given that one of the primary factors in the development of human obesity is enhanced motivation to consume food, these findings are particularly germane to the development of new selective medications to abate the obesity epidemic.

## FUNDING AND DISCLOSURE

The research was funded by Wellcome Trust (WT098012) to LKH; and National Institute of Health (DK056731) and the Marilyn H. Vincent Foundation to MGM. The University of Michigan Transgenic Core facility is partially supported by the NIH-funded University of Michigan Center for Gastrointestinal Research (DK034933). The remaining authors declare no conflict of interest.
