# Supplementary material for: Activation of Ventral Tegmental Area 5-HT2C Receptors Reduces Incentive Motivation
Source: Neuropsychopharmacology. 2016 Dec 21;42(7):1511–21. doi: 10.1038/npp.2016.264 (PMC5362069; doi:10.1038/npp.2016.264)
Supplement: Supplementary Material [file npp2016264x1.docx]

activation of ventral tegmental area 5-ht_2c_ receptors reduces incentive motivation

Lourdes Valencia-Torres^1^, Cristian M. Olarte-Sánchez^1^, David J. Lyons^1^, Teodora Georgescu^1^, Megan Greenwald-Yarnell^2^, Martin G. Myers, Jr^2^, Christopher M. Bradshaw^3^, Lora K. Heisler^1^

^1^ Rowett Institute of Nutrition and Health, University of Aberdeen, Aberdeen, AB21 9SB,UK.

^2^ Division of Metabolism, Endocrinology, and Diabetes, Department of Internal Medicine, University of Michigan, Ann Arbor, MI, USA.

^3^ Division of Psychiatry, University of Nottingham, Queen’s Medical Centre, Nottingham, NG7 2UH, UK.

**Supplementary Materials and Methods**

***Subjects***

A *5-HT_2C_R^CRE^*mouse strain in which Cre recombinase is driven by a 5-HT_2C_R promoter (Burke*et al*, 2016) was intercrossed with a *ROSA26-stop-EYFP* (Enhanced Yellow Fluorescent Protein (EYFP)) reporter mouse line (B6.129X1-Gt(ROSA)26Sortm1(EYFP)Cos/J) to generate a *5-HT_2C_R**^CRE:YFP^* mice. A Cre recombination event excises the tp-A sequence, thereby allowing EYFP to be expressed under the constitutively active ROSA26 locus selectively in 5-HT*_2C_*R*^CRE^* expressing neurons. Experiments were performed on *5-HT_2C_R^CRE:YFP^* adult male and female mice (16–20 weeks at the start of the experiment). As there was no significant effect of sex on the expression of 5-HT_2C_R *^CRE:YFP^* neurons, (n = 6; Student’s t-test; t(4)=0.48, p=0.65), sex-groups were pooled for analysis. Mice were housed individually under a constant light/dark cycle (light on 0600–1800 h). PR performance was assessed during two conditions: ad libitum and food restricted. During the food restricted condition, mice were maintained at 85-90% of their initial free-feeding body weights by providing a limited amount of standard rodent diet after each experimental session. During ad-libitum condition, mice had free access to food in the home cage. Tap water was available in the home cage and environmental enrichment was provided. All experiments were in accordance the U.K. Animals (Scientific Procedures) Act 1986.

***Stereotaxic viral vector injection***

In a stereotaxic apparatus, *5-HT_2C_R^CRE:YFP^* mice were injected bilaterally at a rate of ~0.05 μl min^-1^ with 0.25 µl AAV-hSyn-DIO-hM3D(Gq)-mCherry, AAV-hSyn-DIO-hM4D(Gi)-mCherry or AAV-DIO-mCherry (University of North Carolina Vector Core Facilities, Chapel Hill, NC) into the VTA using a Hamilton syringe (stereotaxic coordinates, mm from bregma: antero-posterior -3.16 ; medio-lateral ±0.56; dorso-ventral -4.5) under 1-3% isoflurane in oxygen anaesthesia. Following injection, the syringe was left in place for 5 min to limit diffusion into the injection track. After removal from the stereotaxic apparatus, mice were monitored for full recovery.

***Operant conditioning***

Two weeks before starting the experiment, the food deprivation regimen was introduced and mice were gradually reduced to 85% of their free-feeding body weights. Mice were trained in operant chambers (MED Associates, Inc.) housed in sound-attenuating boxes and controlled by personal computers using the Med-PC-IV programming language (MED Associates, Inc.). Chambers measured 21.6 cm long × 17.8 cm wide × 12.7 cm high and had a retractable lever, a pellet receptacle, and a 3-W house-light on the opposite wall. Mice were trained to press the lever for 20 mg chocolate pellet reinforcers (TestDiet, St. Louis, MO) and were then exposed to a fixed ratio (FR) 1 schedule for 3 days followed by FR 5 for a further 3 days. Then they underwent daily training under a PR schedule based on an exponential progression derived from the formula (5 × e^0.2n^)−5, rounded to the nearest integer, where n is the position in the ratio sequence (Richardson and Roberts, 1996). At the start of each session, the lever was inserted into the chamber; the session was terminated by withdrawal of the lever 50 min later. Sessions took place at the same time each day during the light phase of the daily cycle (between 0800 and 1300 h) 6 days a week.

The breakpoint was defined as the last ratio completed before 5 min elapsed without any responding or, when this criterion was not met within the session, the highest completed ratio (Olarte-Sánchez *et al*, 2012).

***Mathematical Model (Bradshaw and Killeen, 2012)***

The mathematical model (Bradshaw and Killeen, 2012) used to analyse PR performance was derived from Killeen’s (1994) general theory of schedule-controlled behaviour, the Mathematical Principles of Reinforcement (MPR). MPR is a theoretical account that describes the way in which reinforcers exert control over operant behaviour. The theory is based on three principles: (1) reinforcers activate behavior; (2) There are biological constraints that limit the rate at which animals respond; and (3) The strength of association between responses and reinforcers. These principles are represented by different parameters in the model. For instance, the parameter a (‘specific activation’) is defined as the duration of behavioral activation induced by a single reinforcer and is regarded as an index of incentive value. δ is the minimum time needed to execute a response (the reciprocal of the maximum response rate), and is regarded as a measure of the biological limitations on responding.

The mathematical model (Bradshaw and Killeen, 2012) used in the current manuscript comprises three key equations. The parameters of these equations provide separate numerical indices of the motivational impact of the reinforcer and the motor capability of the organism. The linear waiting equation (Wynne *et al*, 1996) is used to predict the post-reinforcement pause in each ratio (*T*_P,_*_i_*) from the time taken to complete the preceding ratio (total time, *T*_TOT,_*_i‑_*_1_):

*T*_P_*_,i_* = *T*_0_ + *k T*_TOT,_*_i_*_-1_, [1]

where *T*_0_ is the initial post-reinforcement pause and *k* is the slope of the linear waiting function. Two further equations define running response rate, *R*_RUN_, and overall response rate, *R*_OVERALL_, in successive ratios of the schedule:

 [2]

 . [3]

The parameters δ and *a* are the fundamental ‘motor’ and ‘motivational’ parameters of the model: δ expresses the minimum time needed to execute a response, and *a* the duration of behavioural activation induced by a single reinforcer (Bradshaw and Killeen, 2012; Killeen, 1994). Equations 2 and 3 were fitted to the running and overall response rate data obtained from individual mice. Estimates of the parameters, *T_0_*, *k*, *a* and δ, were derived using the ‘Solver’ facility of Excel (Microsoft Corporation); goodness of the combined fit of Eqs. 2 and 3 and the linear relation between post-reinforcement pause duration and the preceding inter-reinforcer interval (cf. Eq. 1) was assessed by *R*^2^ (Bradshaw and Killeen, 2012).

***Immunohistochemistry***

For *c-fos* immunoreactivity (FOS-IR), mice were treated with vehicle, lorcaserin (7 or 10 mg/kg), or CNO (2 mg/kg) i.p. and 90 min later were deeply and terminally anesthetized and then perfused transcardially with 0.9% NaCl, followed by 4% paraformaldehyde (PFA). Brains were extracted and post-fixed in 4% PFA for 4 h, and then dehydrated in 30% sucrose. Brains were sectioned (25 μm) on a freezing microtome and collected in 5 equal series.

Tissue was blocked with 0.3% H_2_O_2_ for 30 min followed by 0.25 % Triton X-100 solution containing 5% normal donkey serum at room temperature for 1 h. Sections were incubated overnight using a rabbit anti-*c-fos* antibody (#2672548, 1:5000, Millipore), followed by Biotin-SP-conjugated donkey anti-rabbit for 90 min (#113772, 1:1000, Jackson ImmunoResearch), avidin-biotin-horseradish peroxidase complex for further 90 min (Vector Laboratories), and visualized with diaminobenzidine solution (DAB peroxidase substrate kit; Vector Laboratories). Immunofluorescence (IF) for 5-HT_2C_R, GAD67, TH, *c-fos*, m-Cherry and green fluorescent protein (GFP) was also performed using a similar procedure, except that the blocking step consisted only of 0.25 % Triton X-100 solution containing 5% normal donkey serum and different primary and secondary antibodies were used. For 5-HT_2C_R, sections were incubated with a mouse monoclonal anti-5-HT_2C_R antibody for 48 h (#L1813, 1:300; Santa Cruz Biotechnology) followed by Alexa Fluor 568 donkey anti-mouse for 1 h (#989784; 1:800, Life Technologies). For c-fos immunofluorescence (FOS-IF), sections were incubated with a rabbit anti-c-fos antibody overnight (#L1610; 1:800, Santa Cruz Biotechnology) followed by Alexa Fluor 568 donkey anti-rabbit for 1 h (#1235798; 1:800, Life Technologies). For GAD67, sections were incubated with a rabbit anti-GAD 67 antibody for 48 h (#J2804; 1:150, Santa Cruz Biotechnology) followed by Alexa Fluor 568 donkey anti-rabbit for 1 h (1:800, Life Technologies). For TH, sections were incubated with a mouse anti-TH antibody (#2716631; 1:1000, Millipore) followed by Alexa Fluor 568 donkey anti-mouse for 1 h (#989784; 1:800, Life Technologies). For m-Cherry, sections were incubated with a rabbit anti-red fluorescent protein overnight (#31089; 1:800, Rockland) followed by Alexa Fluor 568 donkey anti-rabbit for 1 h (#1235798; 1:800, Life Technologies). GFP neurons were visualized using a chicken polyclonal antibody to GFP overnight (#GR279236-1; 1: 800, Abcam) followed by Alexa Fluor 488 donkey anti-chicken for 1 h (#113085; 1:800, Life Technologies).

Images were acquired using an Axioskop II microscope (Carl Zeiss), processed with Adobe Photoshop (Adobe Systems Software, Ireland) and analysed with Image J software (NIH). For IHC quantification analysis, the VTA was defined using the Mouse Brain Atlas (Paxinos and Franklin, 2001) and sections containing the VTA (from bregma -2.92 to -3.88 mm) were counted bilaterally. Values are expressed as mean ± S.E.M. labelled cells per bregma level and analysed using one-factor analyses of variance followed by Tukey’s post hoc comparison.

***Electrophysiology***

For electrophysiological validation, 8-12 week old *5-HT_2C_R^CRE:YFP^* mice were anaesthetized with sodium pentobarbital (Euthatal) and decapitated. The brain was rapidly removed and placed in cold (0-4^o^C), oxygenated (95%O_2_/5%CO_2_) ‘slicing’ solution containing (in mM) sucrose (214)**,** KCl (2.5), NaH_2_PO_4_ (1.2), NaHCO_3_ (26), MgSO_4_ (4), CaCl_2_ (0.1), D-glucose (10). The brain was glued to a vibrating microtome (Campden Instruments, Loughborough, UK) and 200μm thick coronal sections containing the VTA were prepared. Slices were immediately transferred to a ’recording’ solution containing (in mM) NaCl (127), KCl (2.5), NaH_2_PO_4_ (1.2), NaHCO_3_ (26), MgCl_2_ (1.3), CaCl_2_ (2.4), D-glucose (10), in a continuously oxygenated holding chamber at 35ºC for a period of 25 min. Subsequently, slices were allowed to recover in ‘recording’ solution at room temperature for a minimum of 1h before recording. For whole-cell recordings, slices were transferred to a submerged chamber and a Slicescope upright microscope (Scientifica, Uckfield, UK) was used for infrared - differential interference contrast and fluorescence visualization of cells. During recording, unless otherwise described, slices were continuously perfused at a rate of ca. 2 ml/min with oxygenated ‘recording’ solution (as above) at room temperature. All pharmacological compounds were bath applied. Whole cell current- and voltage-clamp recordings were performed with pipettes (3-7MΩ when filled with intracellular solution) made from borosilicate glass capillaries (World Precision Instruments, Aston, UK) pulled on a Zeitz DMZ micropipette puller (Zeitz Instruments GmBH, Martinsried, Germany). The intracellular recording solution contained (in mM) K-gluconate (140), KCl (10), HEPES (10), EGTA (1), Na_2_ATP (2), pH 7.3 (with KOH). Recordings were performed using a Multiclamp 700B amplifier and pClamp10 software (Molecular Devices, Sunnyvale, CA, USA). Liquid junction potential was 16.4mV and not compensated. The recorded current was sampled at 10 kHz and filtered at 2 kHz unless otherwise stated.

**Supplementary Figures**


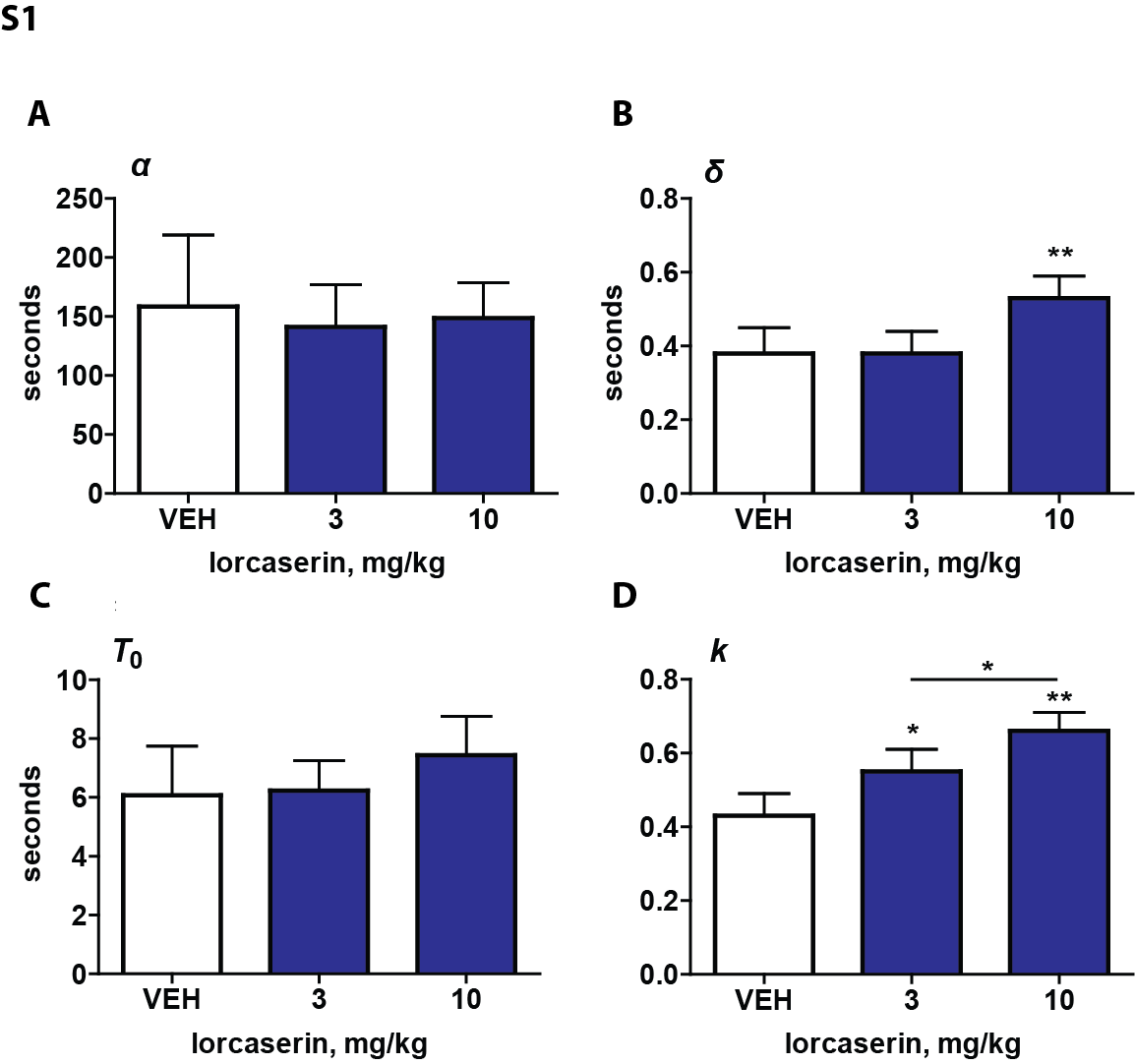


***Figure S1. Effect of lorcaserin on parameters of the progressive-ratio model*.** (A) Lorcaserin 3 and 10/mg kg had no effect on the ‘motivational’ parameter, *a* (n = 9; repeated-measures one-way ANOVA; F(2,16)=0.31; N.S.) (B) The ‘motor’ parameter, δ, was increased by lorcaserin 10 mg/kg (n = 9; repeated-measures one-way ANOVA; (F(2,16)=19.6; p<0.0001; followed by Dunnett’s test). (C) There was no effect on *T_0_* (n = 9; repeated-measures one-way ANOVA; F(2,16)=0.93; N.S.). (D) Lorcaserin 3 and 10 mg/kg significantly increased the value of *k* (n = 9; repeated-measures one-way ANOVA; F(2,16)=14.46; *p*<0.0001; followed by Dunnett’s test). All data are presented as mean ± SEM. ∗p<0.05; ∗∗p < 0.01; ∗∗∗p < 0.001.


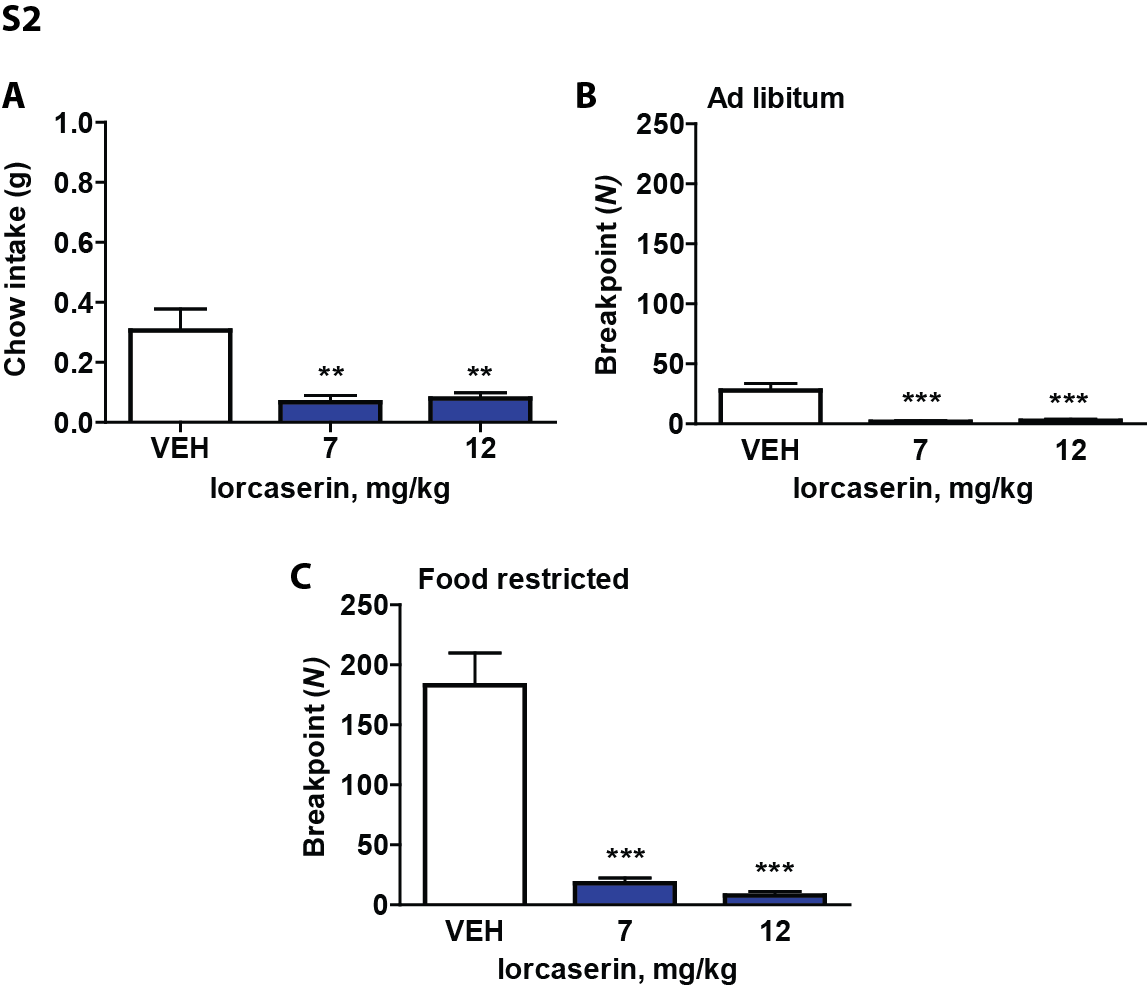


***Figure S2. Lorcaserin reduces chow intake and progressive ratio breakpoint.*** (A) Lorcaserin (7 and 12 mg/kg) reduced dark cycle home cage chow intake in the ad libitum fed condition (n = 8-9 per group; one-way ANOVA; F(2,19)=8.12; p<0.001; with Tukey’s post hoc comparison), and (B) reduced progressive ratio responding breakpoint in the ad libitum fed condition (n = 25; repeated-measures one-way ANOVA; F(2,48)=41.65; p<0.0001; followed by Dunnett’s test) and (C) food restricted condition (n = 25; repeated-measures one-way ANOVA; F(2,48)=23.58; p<0.0001; followed by Dunnett’s test). Data are presented as mean ± SEM. ∗∗p < 0.01; ∗∗∗p < 0.001.


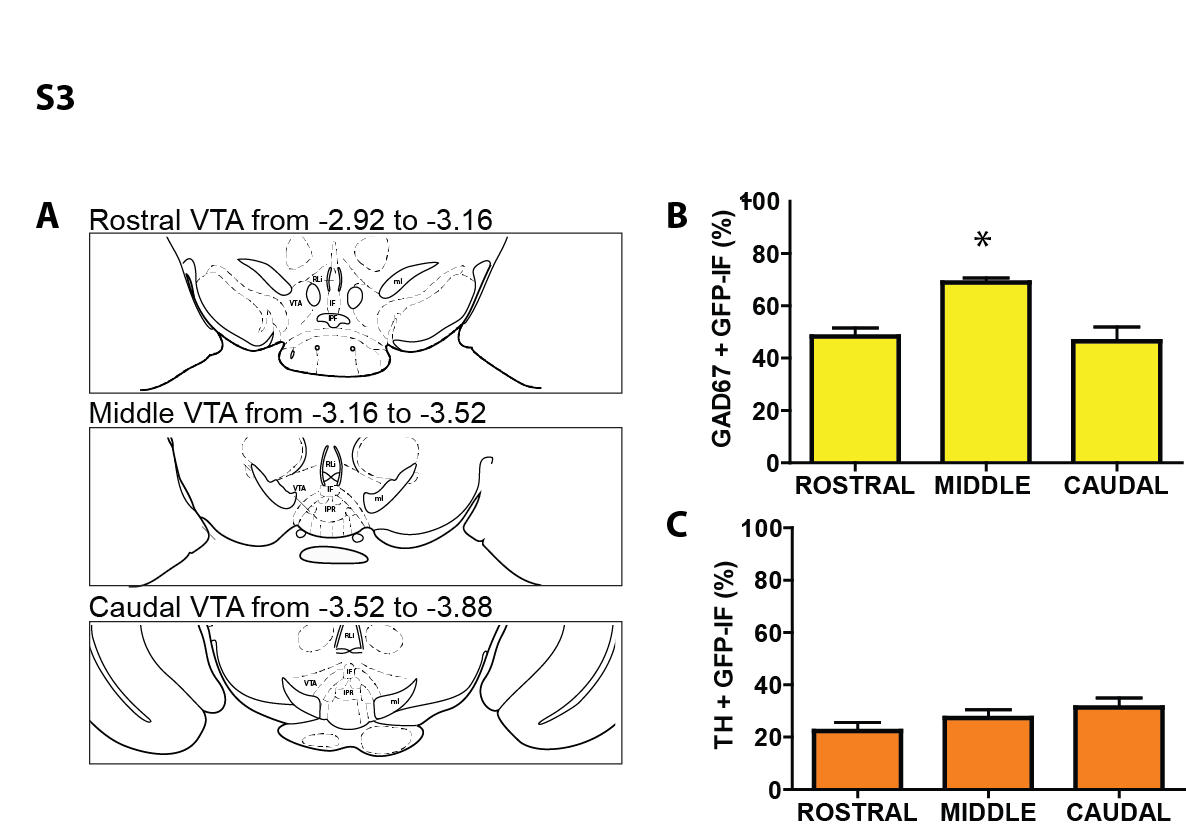


***Figure S3. Percentage of*** ***5-HT_2C_ receptor expressing neurons that co-express GAD67 and TH*.** (A) Schematic representing the location of the VTA in a mouse brain (Paxinos and Franklin, 2001) where the cells were counted. (B) Percentage of 5-HT_2C_ receptor expressing neurons that co-express GAD67 in different bregma levels of the VTA. There was a higher percentage of co-localization in the middle level compared to the rostral and caudal levels (n = 3 per group; one-way ANOVA; F(2,6)=10.65; *p*<0.05; followed by Tukey’s post hoc test). (C) Percentage of 5-HT_2C_ receptor expressing neurons that co-express TH in different bregma levels of the VTA. There was no significant difference on the percentage of co-localization in the different VTA bregma levels (n = 3 per group; one-way ANOVA; F(2,6)=1.84, *N.S* ). All data are presented as mean ± SEM. ∗p<0.05; ∗∗p < 0.01; ∗∗∗p < 0.001.


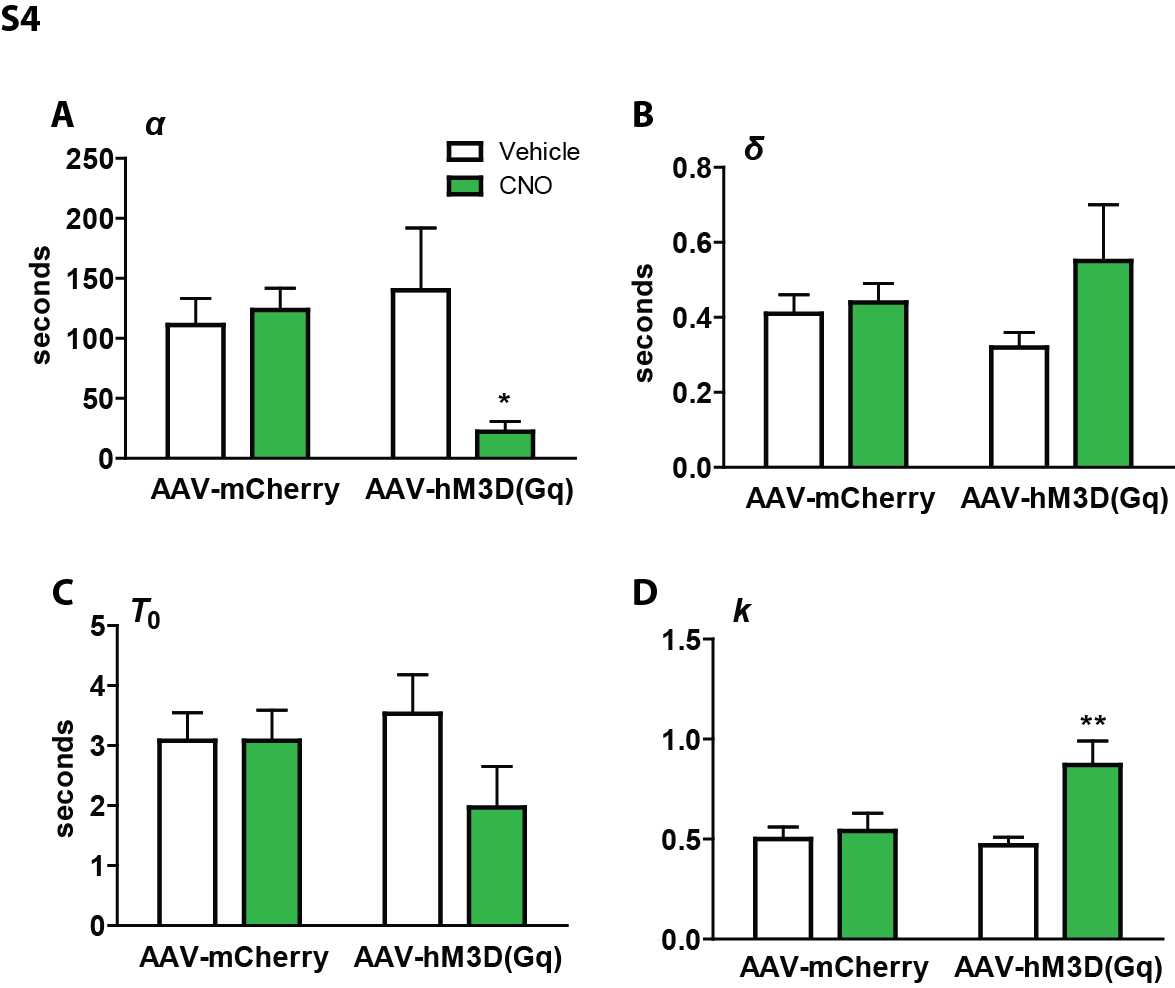


***Figure S4. Effect of chemogenetic activation of VTA 5-HT_2C_R neurons on parameters of the progressive-ratio model*.** (A) CNO 2mg/kg reduced the ‘motivational’ parameter, *a*, in the 5-HT_2C_R^CRE:YPF^::hM3D_q_-mCherry^VTA^ mice (n = 15; two-factor ANOVA with repeated measures on treatment; F(1,13)=6.6; p<0.05; with Tukey’s post-hoc comparison). (B) There was no effect on the ‘motor’ parameter, δ, (n = 15; two-factor ANOVA with repeated measures on treatment; F(1,13)=1.7; N.S). (C) Activation of VTA 5-HT_2C_Rs neurons had no effect on *T*_0_ (n = 15; two-factor ANOVA with repeated measures on treatment; F(1,13)=1.3; *N.S.*). (D) CNO 2mg/kg significantly increased the value of *k* in the 5-HT_2C_R^CRE:YPF^::hM3D_q_-mCherry^VTA^ mice (n = 15; two-factor ANOVA with repeated measures on treatment; F(1,13)=12.6, *p<*0.01; with Tukey’s post-hoc comparison). All data are presented as mean ± SEM. ∗p<0.05; ∗∗p < 0.01; ∗∗∗p < 0.001.
